# Evolution of Femoral Cannulation Techniques in Minimally Invasive Mitral Valve Surgery: A 10-Year Experience

**DOI:** 10.3390/medsci14020182

**Published:** 2026-04-03

**Authors:** Jonah Schwarz, Parwis Massoudy, Marius Mihai Harpa, Markus Czesla, Christian Mogilansky, Klara Brînzaniuc, Emanuel-David Anitei, Robert Balan

**Affiliations:** 1 Department of Cardiac Surgery, Klinikum Passau, 94032 Passau, Germany; jonah.schwarz@klinikum-passau.de (J.S.); parwis.massoudy@klinikum-passau.de (P.M.); markus.czesla@klinikum-passau.de (M.C.); christian.mogilansky@klinikum-passau.de (C.M.); 2 Department of Surgery IV, George Emil Palade University of Medicine, Pharmacy, Science and Technology of Targu Mures, 540142 Targu Mures, Romania; 3 Department of Cardiovascular Surgery, Emergency Institute for Cardiovascular Diseases and Transplantation Targu Mures, 540136 Targu Mures, Romania; 4 Department of Anatomy and Embryology, George Emil Palade University of Medicine, Pharmacy, Science and Technology of Targu Mures, 540142 Targu Mures, Romania; klara_branzaniuc@yahoo.com; 5 Doctoral School, George Emil Palade University of Medicine, Pharmacy, Science and Technology of Targu Mures, 540142 Targu Mures, Romania; anitei_emanuel@yahoo.com (E.-D.A.); balanrobert2003@gmail.com (R.B.)

**Keywords:** minimally invasive surgery, percutaneous cannulation, mitral valve surgery

## Abstract

**Background:** Femoral cannulation is essential for minimally invasive mitral valve surgery (MIMVS). Our center transitioned from open femoral cut-down to ultrasound-guided percutaneous cannulation supported by smart venous cannulas, ThruPort arterial access, and the MANTA closure device. This study evaluates how this transition affects procedural efficiency, vascular safety, and postoperative outcomes. **Methods:** We retrospectively analyzed 575 consecutive MIMVS patients (2014–2025). Patients treated before 2021 formed the cut-down group, while those from 2021 onward underwent percutaneous cannulation. The outcomes included operative times, groin and lymphatic complications, MANTA performance, and 30-day mortality. Propensity score matching (PSM) was performed to adjust for baseline differences. **Results:** Of 575 patients, 393 (68.3%) underwent cut-down and 182 (31.7%) percutaneous access. Percutaneous access was associated with shorter cardiopulmonary bypass times (115 vs. 128 min, *p* < 0.0001), total operative times (210 vs. 242 min, *p* < 0.0001), ICU stays (2 vs. 3 days, *p* = 0.0267), and hospital stays (8 vs. 11 days, *p* < 0.0001). Lymph fistula occurred in 4.3% of cut-down cases and in 0% after the adoption of percutaneous access (*p* = 0.0004). Overall groin complication rates were comparable (2.8% vs. 4.9%, *p* = 0.51). MANTA closure had a 2.2% device-related complication rate (1.1% bleeding; 1.1% ischemia) with no documented long-term sequelae. Regarding 30-day mortality, this was 4.6% in the cut-down group and 1.6% in the percutaneous group (*p* = 0.096). In PSM (72 matched pairs), percutaneous access retained significantly shorter operative, bypass, and ICU times, with identical groin complication rates. **Conclusions:** Ultrasound-guided percutaneous femoral cannulation was associated with improved procedural efficiency and elimination of lymphatic morbidity, without increasing vascular risk or mortality. It represented a safe and effective standard strategy for contemporary MIMVS.

## 1. Introduction

Minimally invasive mitral valve surgery (MIMVS) has evolved into a well-established approach for treating mitral valve disease [1,2,3,4]. Early work by Cohn et al. [5] demonstrated that limited access valve surgery improved patient satisfaction. Subsequent meta-analyses and registry studies confirmed comparable outcomes to sternotomies while minimizing trauma [6,7,8,9,10,11]. A key part of this surgery has remained the establishment of safe femoral access of the heart–lung machine for cardiopulmonary bypass (CPB). The traditional method of an open femoral cut-down of the femoral artery and vein has been effective; however, it can prolong procedures and increase risks of lymphatic or wound complications. The rise in large-bore percutaneous access for transcatheter interventions [12,13,14,15] has led cardiac surgeons to adopt similar methods. The introduction of ultrasound guidance, smart venous cannula, and plug-based closure systems such as MANTA [13,14,16,17,18,19] have enabled percutaneous cannulation and decannulation with reduced invasiveness. Pausch et al. [20] and recent meta-analyses [21] have evaluated the safety and efficiency of different vascular closure devices for percutaneous cannulation in a large multicenter registry; however, these did not look at the differences between percutaneous cannulation and cut-down cannulation in MIMVS. Despite the increasing use of percutaneous cannulation in transcatheter and endovascular procedures, its role in minimally invasive mitral valve repair is still evolving. Many centers have remained cautious due to concerns regarding arterial safety under full heparinization, the implementation of new techniques associated with ultrasound guidance, and the uncertainty surrounding large-bore closure device performances in surgical patients. Most published evidence has consisted of single-surgeon series or short adoption windows, which cannot reflect the full institutional learning curve [22,23,24,25]. A key unresolved question regarding whether efficiency gains are achieved without compromising quality has remained. By examining outcomes over a full decade of program development, our study captured the transition from early experience to procedural maturity, allowing the assessment of how cannulation strategy can influence operative workflow, complication profiles, and the capability to perform complex reconstructive mitral repairs.

Our center’s decade-long experience and this institutional transition can provide an opportunity to examine this progression in real-world practice, where patient selection has broadened and surgical experience has deepened over time. By analyzing all patients treated during the learning period rather than in selected sub-cohorts, this study captured the institutional evolution from routine cut-down cannulation to a standardized percutaneous approach. This allowed the evaluation not only of procedural safety, but also of workflow efficiency, resource utilization, and the maturing complexity of mitral valve repairs performed.

This study summarized our 10-year experience, comparing traditional surgical cut-down with modern percutaneous femoral cannulation in terms of procedural times, vascular complications, and early outcomes. We hypothesized that percutaneous access would improve efficiency and reduce leg-related morbidity without compromising safety.

## 2. Materials and Methods

### 2.1. Study Design and Population

This retrospective single-center analysis included all patients that underwent minimally invasive mitral valve surgery during the study period between January 2014 and August 2025 at the Department of Cardiac Surgery, Klinikum Passau, Germany. The study protocol was approved by the ethics committee of the University of Regensburg, and all data were pseudonymized. We included all minimally invasive mitral valve procedures performed via a right mini-thoracotomy, including isolated mitral valve repairs or replacements and combined procedures (e.g., tricuspid valve repair, atrial septal defect closure, surgical atrial fibrillation ablation). Cases in which a primary non-mitral procedure was performed were excluded.

Patients were divided into two groups according to cannulation strategy: the cut-down group included all procedures performed before January 2021 using open surgical femoral exposure, whereas the percutaneous group included all procedures performed from January 2021 onward using documented ultrasound-guided percutaneous femoral cannulation. Patients with incomplete cannulation or operative data were excluded. All patients considered for MIMVS underwent routine preoperative CT angiography to assess the suitability of the femoral vessels for peripheral cannulation. The main criteria used to exclude peripheral femoro–femoral access were a femoral artery diameter < 6 mm, anterior calcification at the intended cannulation site, and aortic thrombosis. If one or more of these findings were present, the procedure was performed via a full sternotomy.

### 2.2. Surgical Technique

All procedures were performed through a right anterolateral mini-thoracotomy in the fourth intercostal space. Cardiopulmonary bypass was established through peripheral femoro–femoral cannulation. In the cut-down period, the femoral artery and vein were exposed through a small groin incision. After total preparation of the groin vessels, vascular loops were placed around the femoral vessels. Two purse-string sutures with a tourniquet were routinely used for the arterial cannulation site and one purse-string suture for the venous one. Cannulation was performed using the Seldinger technique under direct vision and repaired by knotting the purse-string sutures after decannulation.

The percutaneous technique employed an ultrasound-guided puncture of the femoral artery and vein. First, the femoral vein was punctured, and after introducing the guide wire, its position was secured in the superior vena cava with a transesophageal echocardiography (TEE). Before the dilators and the cannula were introduced, a second puncture was performed for the femoral artery in order to avoid ultrasound artifacts. Either a 24 Fr. Smart Venous Cannula (SmartCanula™, Lausanne, Switzerland) or a multistage venous cannula (23 Fr Bio-Medicus, Medtronic, Minneapolis, MN, USA) was used for venous drainage, and the 21–23 Fr. ThruPort™ cannula (Medtronic, Minneapolis, MN, USA) was used for arterial access.

For arterial decannulation, the MANTA vascular closure device (Teleflex, Wayne, PA, USA) was implanted for hemostasis. For venous decannulation, a Z-suture was placed, and constant pressure was applied with a Safeguard^®^ compression device (Merit Medical Systems, South Jordan, UT, USA). Cardiopulmonary bypass was established with moderate hypothermia (34 °C). In the majority of cases, an aortic cross clamp was performed with the use of an intra-aortic balloon, and antegrade cold blood cardioplegia were routinely used—we opted for a single-shot Bretschneider cardioplegic solution. Generally, 1800 mL of cardioplegic solution was applied over a period of six minutes through the cardioplegic branch of the EndoClamp-Balloon [26,27].

### 2.3. Data Collection and Variables

Data were extracted from the institutional surgical database and reviewed manually. These variables included baseline characteristics: age, sex, obesity (BMI > 30 kg/m^2^), EuroSCORE II, preoperative NYHA class, COPD, chronic dialysis, and prior cardiac surgery (any previous commissurotomy, harpoon, mitral valve replacement, bypass, aortic valve replacement, or prior sternotomy). The operative variables included: cardiopulmonary bypass time, aortic cross-clamp time, total operation time, ICU stay, hospital stay, and the need for re-exploration for bleeding. The outcomes from the access site and device included: groin complications, lymph fistula, wound healing disorders, the use of MANTA closure, MANTA-related complications (ischemia or bleeding). Mortality was between <30 days and >30 days. Groin complications were defined as a composite endpoint, including access site hematoma requiring intervention, pseudoaneurysm, arterial or venous thrombosis, limb ischemia, access site infection, seroma, or the need for surgical or endovascular intervention. Lymph fistula and wound-healing disorders were analyzed separately, as lymphatic injury represented a distinct mechanism related to surgical dissection.

### 2.4. Statistical Analysis

The primary endpoint was the occurrence of groin complications. Secondary endpoints included the cardiopulmonary bypass time, total operative time, ICU length of stay, hospital length of stay, re-exploration for bleeding, and 30-day mortality. The re-exploration for bleeding was analyzed descriptively and was not further evaluated in a separate multivariable model.

As continuous variables demonstrated a non-normal distribution, they were expressed as a median and an interquartile range (IQR). Categorical variables were presented as counts and percentages. The continuous variables were compared using the Mann–Whitney U test. The categorical variables were compared using Fisher’s exact or chi-square tests, as appropriate. A two-sided *p* < 0.05 was considered statistically significant. After propensity score matching, the outcomes were compared using paired analyses. To account for the residual imbalance after matching, a multivariable linear regression model with standard errors clustered by the matched pair was fitted for the ICU length of stay, including the treatment group, NYHA class, COPD, age, and obesity. Because the ICU length of stay showed a skewed distribution, this variable was log-transformed before regression modeling. Accordingly, the regression coefficient reflected an effect on the log scale and was not directly comparable to the median difference reported in the descriptive analyses. The analyses were performed using Python 3.13 (Python Software Foundation, Beaverton, OR, USA), with the pandas 3.0.2 and SciPy 1.15.3 libraries (NumFOCUS, Austin, TX, USA).

### 2.5. Propensity Score Matching

To reduce a potential confounding bias, we performed propensity score matching (PSM) comparing percutaneous versus cut-down cannulation. The probability of receiving percutaneous cannulation was estimated using a multivariable logistic regression model and included the following preoperative covariates: age, sex, obesity (BMI > 30), EuroSCORE II, NYHA class, COPD, chronic dialysis, and history of previous cardiac surgery.

Matching was performed using nearest neighbor matching in a 1:1 ratio, without replacements. The caliper width was 0.2 of the standard deviation of the logit of the propensity score. The covariate balance before and after matching was assessed using standardized mean differences, with an absolute value of < 0.10 considered indicative of an adequate balance.

Because propensity score matching resulted in paired observations, continuous outcomes in the matched cohort were compared using the Wilcoxon signed-rank test, and binary outcomes were compared using McNemar’s test. This approach accounted for within-pair correlation and preserved statistical power.

This analysis was performed with Python (Pandas, Scipy, Scikit-learn). As continuous variables were non-normally distributed, data were presented as a median and an interquartile range (IQR) throughout, including in the matched cohort.

## 3. Results

### 3.1. Patient Population and Baseline Characteristics

A total of 575 patients underwent minimally invasive mitral valve surgery during the study period: 393 (68.3%) in the cut-down group and 182 (31.7%) in the percutaneous group. The baseline demographics are summarized in Table 1.

The median age was slightly higher in the cut-down group (68 [58–76] years) than in the percutaneous group (65 [57–74] years, *p* < 0.05). The proportion of women was comparable (38.9% vs. 32.4%, *p* > 0.05). Obesity was more frequent in the cut-down group (26.7% vs. 17.2%, *p* < 0.01). EuroSCORE II was marginally higher before 2021 (1.48 [0.96–2.56] % vs. 1.21 [0.73–2.51] %, *p* < 0.01). By contrast, NYHA class was similar in both groups (median 3 [2–3] vs. 3 [2–3], *p* = 0.87), as were the rates of COPD (11.7% vs. 7.7%, *p* = 0.20) and chronic dialysis (2.9% vs. 2.7%, *p* = 0.89). Previous cardiac surgery was more common in cut-down patients (10.2% vs. 3.8%, *p* = 0.005).

### 3.2. Surgical Characteristics

Of the different surgeries performed, the number of tricuspid valve reconstructions (10.2% vs. 11%), mitral valve replacements (12.5% vs. 9.9%), and closures of atrial septal defects (22.1% vs. 16.5%) showed no significant differences between both groups. On the other hand, annuloplasties (73% vs. 81.9%), the implantation of neochordae (56.2% vs. 69.8%), and left (21.1% vs. 33%) and biatrial (0% vs. 2.2%) cryomaze ablations increased with time, as seen in Table 2.

Notably, the proportion of patients undergoing combined annuloplasty and neochordae repair increased substantially after the transition to percutaneous cannulation (54.5% vs. 69.8%, *p* < 0.01). This suggested that increasing institutional experience did not lead to the simplification of repairs, but rather it enabled a higher level of reconstructive mitral techniques.

### 3.3. Operative Results

The median cardiopulmonary bypass time was significantly shorter in the percutaneous group (115 [98–135] min vs. 128 [107–163] min, percutaneous vs. cut-down, *p* < 0.01). The cross-clamp time was comparable between groups (75 [60–95] vs. 75 [61–94] min, percutaneous vs. cut-down, *p* > 0.05). The total operation time was reduced by approximately half an hour after adopting percutaneous access (210 [185–248] vs. 242 [211–292] min, percutaneous vs. cut-down, *p* < 0.01). The median hospital stay was shorter in percutaneous cases (8 [7–11] days vs. 11 [8–16] days, *p* < 0.01). The ICU length-of-stay data were available for both groups and are reported in Table 3. The re-exploration for bleeding occurred more often after percutaneous access (12.1%; 95% CI 7.7–17.7) than after cut-down cannulation (4.5%; 95% CI 2.7–7.1; *p* < 0.01), although most re-entries were related to intrathoracic oozing. The operative outcomes are detailed in Table 3.

The reduction in the operative and bypass times occurred despite an increase in the proportion of complex repairs, indicating that the workflow efficiency improved independently of the surgical complexity.

### 3.4. Access Site- and Device-Related Outcomes

**Figure 1:**
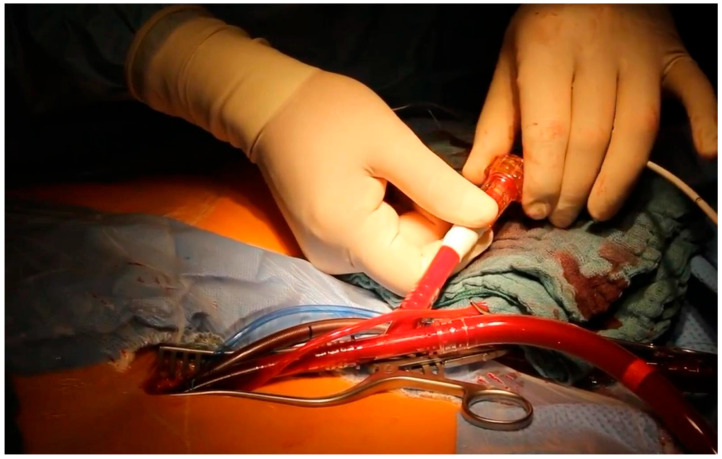
Successful femoral cannulation via cut-down with a Thruport arterial cannula.

The overall groin complication rates were comparable between groups (2.8% vs. 4.9%, *p* = 0.51). Lymph fistula occurred exclusively after open femoral cut-down (4.3% vs. 0%, *p* = 0.0004), as seen in Figure 1.

The MANTA device was used in 97.2% of percutaneous cases. MANTA-related complications occurred in four patients (2.2%; 95% CI 0.6–5.5), including severe bleeding in two patients (1.1%; 95% CI 0.1–3.9) and limb ischemia in two patients (1.1%; 95% CI 0.1–3.9). All four complications required treatment by the vascular surgical team and were associated with prolonged ICU stays. The access site- and device-related outcomes are detailed in Table 4.

### 3.5. Results of the Propensity Score Matching

**Figure 2:**
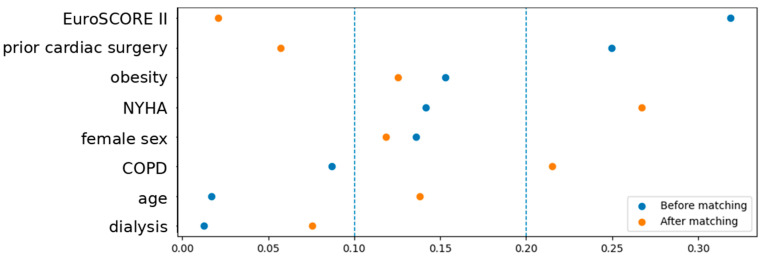
Love plot of covariate balance before and after propensity score matching. Absolute standardized mean differences demonstrate improved balance after matching, with minor residual imbalance for NYHA class and COPD.

Propensity score matching resulted in 72 matched pairs (144 patients), with 431 patients excluded from the original cohort. The covariate balance improved substantially after matching, as demonstrated by standardized mean differences and visualized in the Love plot in Figure 2.

The results of the matched analysis are shown in Table 5. In the matched cohort, the overall groin complication rates were identical between groups (2.8% vs. 2.8%; *p* = 1.00). The cardiopulmonary bypass time (97.0 [82.0–116.0] vs. 104.5 [89.0–123.0] min; *p* = 0.028) and cross-clamp time (68.0 [56.0–83.0] vs. 74.0 [61.0–89.0] min; *p* = 0.041) remained shorter in the percutaneous group, and the total operative time was also reduced (213.0 [191.0–246.0] vs. 244.0 [213.0–272.0] min; *p* = 0.002).

The ICU length of stay was significantly shorter after percutaneous cannulation (2.0 [2.0–3.2] vs. 3.0 [2.0–5.8] days; *p* = 0.016). In the paired analysis using the Wilcoxon signed-rank test, percutaneous access was associated with a median reduction of 0.5 ICU days (*p* = 0.0015).

To account for the residual imbalance after matching, a multivariable linear regression model with standard errors clustered by the matched pair was fitted for the ICU length of stay. After adjusting for NYHA class, COPD, age, and obesity, percutaneous cannulation remained independently associated with a shorter ICU stay in the clustered regression model (β = −3.5; 95% CI −5.5 to −1.6; *p* < 0.001). Because the ICU length of stay was log-transformed before analysis, this coefficient reflected an effect on the log scale and should not be interpreted as an absolute reduction in the ICU days.

## 4. Discussion

This 10-year single-center experience suggested that the transition from surgical cut-down to percutaneous femoral cannulation in minimally invasive mitral valve surgery (MIMVS) was safe and was associated with improved procedural metrics and fewer lymphatic complications. However, because percutaneous cannulation was adopted later in the study period, part of the observed gains may also reflect institutional maturation, including increasing surgical experience, refinements in perioperative management, and a progressive standardization of minimally invasive workflows.

### 4.1. Procedural Efficiency

**Figure 3:**
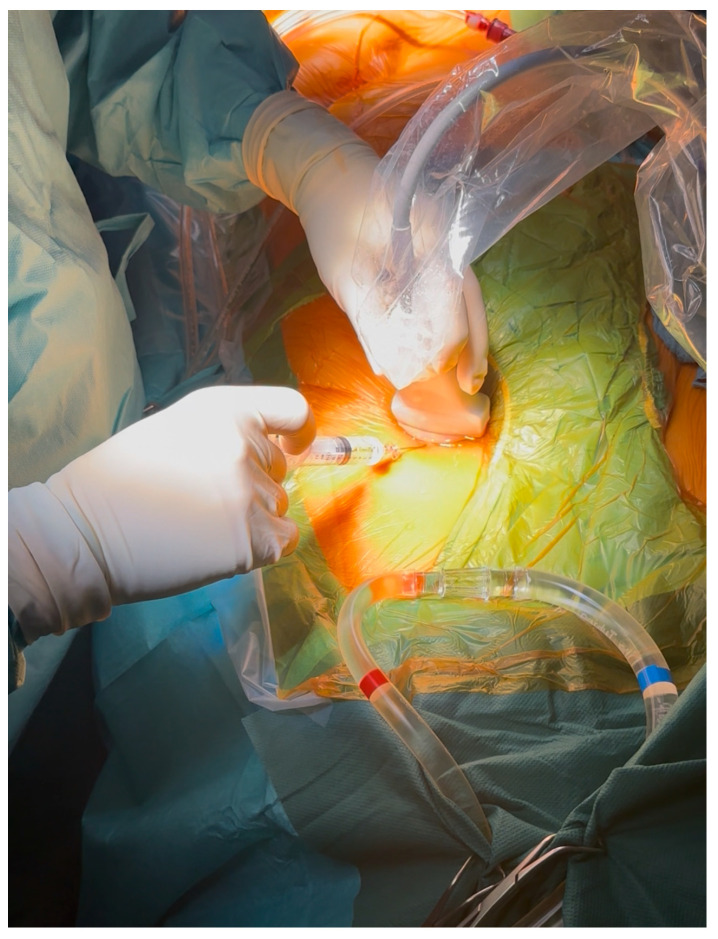
Ultrasound-guided percutaneous cannulation of the right femoral artery and vein.

**Figure 4:**
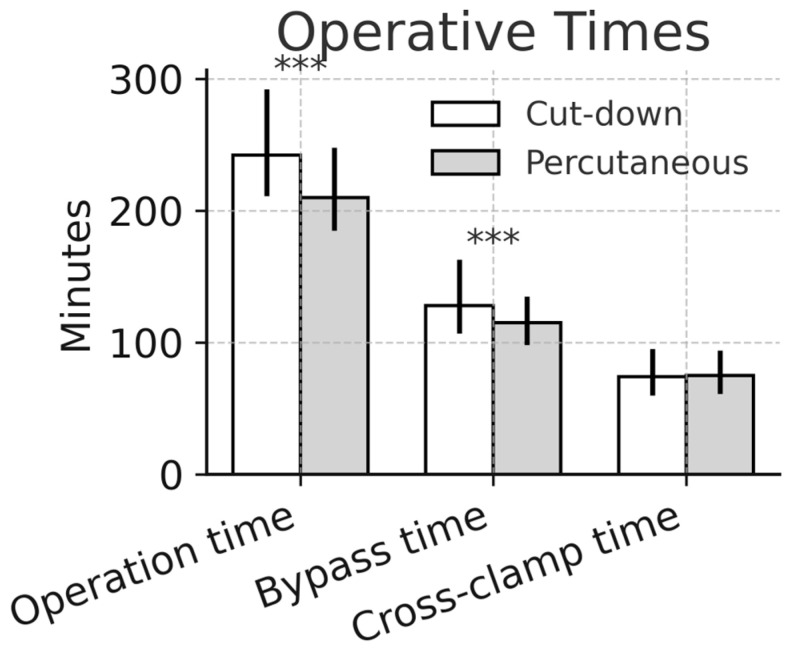
Comparison between cut-down and percutaneous cannulation for total operation time, bypass time, and cross-clamp time. *** for *p* < 0.01.

Percutaneous access significantly shortened incision-to-suture times, as shown in Figure 3. The difference in operative duration persisted after propensity score matching, as illustrated in Figure 4. The approximately 30 min reduction in total operative time likely reflected several cumulative effects, including the elimination of surgical vessel exposure and closure, a faster setup for CPB, and an improved team workflow once the technique had been standardized. This time saving was consistent with prior reports by Pozzi et al. [28] and Saeed et al. [29], who observed similar efficiency gains following the adoption of ultrasound-guided femoral access in minimally invasive and robotic valve surgery [1,2].

There was also a significant difference in the CPB times between the two groups, as shown in Figure 4; however, because vascular access was established before initiation of cardiopulmonary bypass, this finding cannot be attributed solely to the use of percutaneous cannulation. Similarly, these reductions in operative times should not be interpreted as being exclusively related to the cannulation method itself. The cannulation time was not recorded as a separate variable in our database, and therefore, the individual contribution of vascular access cannot be quantified precisely. The shorter operative, bypass, and cross-clamp times observed during the percutaneous period most likely reflected a combination of factors, including the avoidance of surgical vessel exposure and closure, a faster CPB setup, the progressive refinement of surgical technique, and an improved team workflow as the minimally invasive program matured.

This interpretation can be further supported by the case mix. There was no reduction in procedural complexity over time; on the contrary, the proportion of major procedures increased. These findings suggested that the observed reduction in operative times was achieved despite the increasing case complexity while maintaining the institutional standard of care.

### 4.2. Vascular Safety

**Figure 5:**
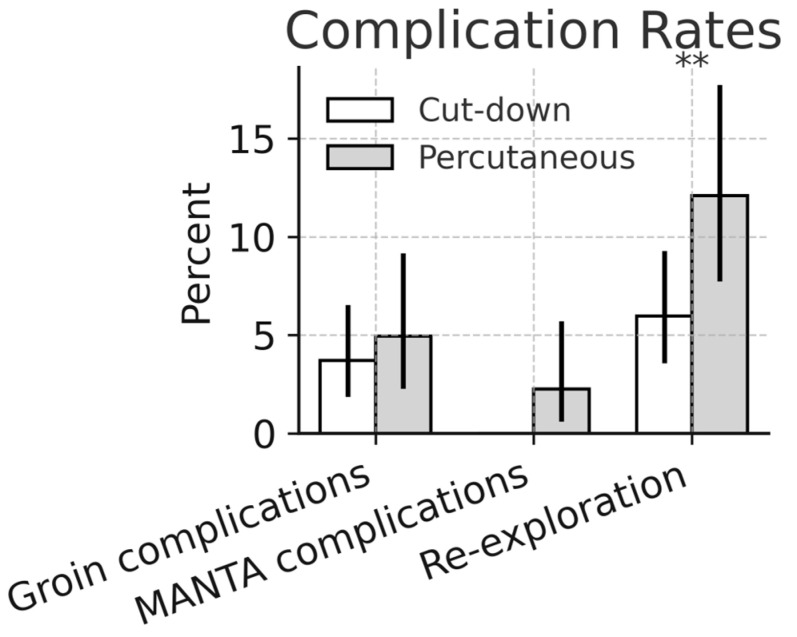
Comparison between cut-down and percutaneous cannulation for groin complications, MANTA complications (only shown for the percutaneous cohort), and re-exploration. ** for *p* < 0.05.

Despite theoretical concerns regarding percutaneous cannulation under systemic heparinization, vascular complication rates remained low and comparable to open femoral cut-down. Overall groin complication rates remained low and comparable between the groups, while lymphatic complications were eliminated after the adoption of the percutaneous strategy [15,19,30]. In the matched cohorts, both forms of access had equal rates of groin complications (2.8%). Our data suggested that percutaneous cannulation was as safe as open femoral cut-down, as shown in Figure 5.

The MANTA closure device proved reliable, with only two minor bleeding events (1.1%) and two ischemic injuries (1.1%). This aligned with the 0–2% complication rate reported for plug-based closure systems in minimally invasive cardiac surgery [16,17,20,31]. Our results reinforced that, with preoperative CT angiography, careful ultrasound guidance, precise vessel sizing, and trained surgeons, percutaneous closure was safe even under full anticoagulation.

### 4.3. Re-Exploration and Bleeding

Re-exploration for bleeding occurred more frequently in the percutaneous group (12.1% vs. 4.5%). Importantly, all re-explorations were due to intrathoracic bleeding, and none were related to groin access or vascular complications. As re-exploration represented a clinically relevant adverse outcome, this finding warranted careful consideration; however, because this endpoint was not evaluated in a dedicated multivariable model, no independent inference can be made regarding its determinants. The observed difference may be related to temporal and procedural factors, including increasing procedural complexity, evolving perioperative anticoagulation strategies, and learning curve effects during the transition period. These explanations should be regarded as clinically plausible but exploratory, rather than independently confirmed. The persistence of shorter ICU and hospital stays despite higher re-exploration rates supported this interpretation; however, given the retrospective design and inherent period effects, a causal relationship cannot be definitively excluded.

### 4.4. Hospital Course and Recovery

The shorter hospital stay (median 8 vs. 11 days) reflected a faster recovery, reduced wound morbidity, and more predictable postoperative management. This finding parallelled broader experience from minimally invasive valve surgery and transcatheter procedures, where reduced access trauma enabled earlier mobilization and discharge [6,7,8,12,31]. Mortality remained low and comparable between the groups, underscoring that percutaneous access did not compromise patient safety.

**Figure 6:**
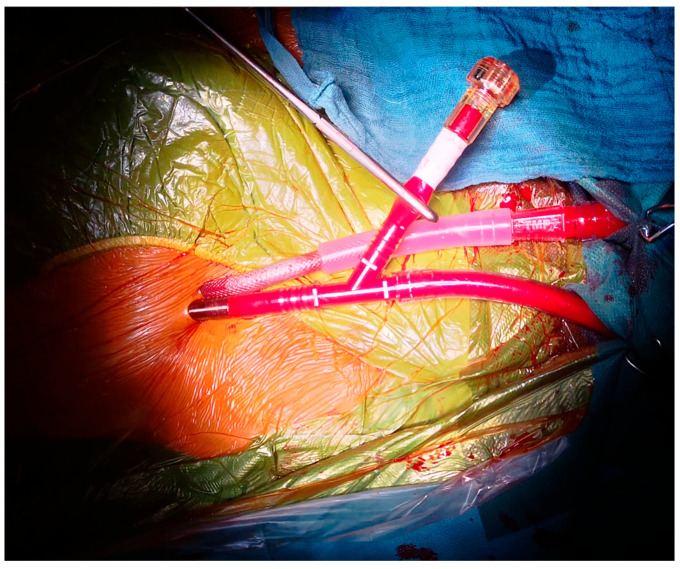
Successful percutaneous cannulation of the right groin with a smart cannula and a Thruport cannula.

**Figure 7:**
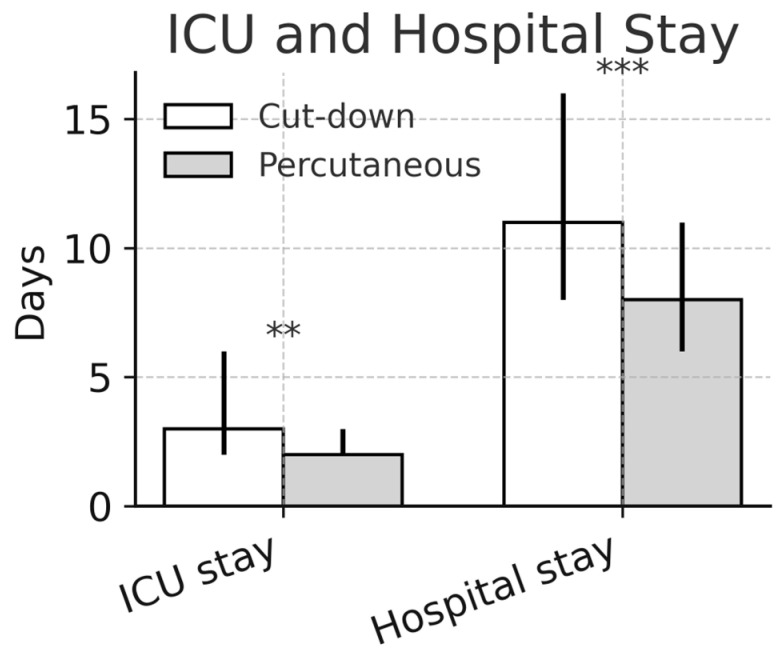
Comparison between cut-down and percutaneous cannulation for the length of stay in the ICU and the total length of stay in the hospital. ** for *p* < 0.05, *** for *p* < 0.01.

After the transition to percutaneous cannulation, the ICU length of stay decreased from 3 to 2 days, as shown in Figure 6 and Figure 7. A similar difference remained after propensity score matching; however, this finding should be interpreted with caution. Because the ICU length of stay was modeled using log-transformed data in the adjusted analysis, the regression coefficient reflected an effect on the transformed scale and was not directly comparable to the descriptive median difference. In addition, the observed reduction in the ICU stay was likely multifactorial and may reflect not only the change in cannulation strategy but also increasing institutional experience, improved perioperative care, and the progressive standardization of the minimally invasive program. Nevertheless, the direction of this finding mirrored the results reported by Schäfer et al. [31], who used a similar setup and reported a mean ICU stay of 1.7 days. Earlier mobilization and reduced groin-related discomfort may have also contributed to the shorter ICU and hospital stays observed after the adoption of the percutaneous strategy.

During the last year of the study period, our center began implementing a fast-track and ERAS (enhanced recovery after surgery) pathway for suitable patients. As this pathway included percutaneous cannulation and extubation in the operating theater, we have been hopeful that we can achieve further reduction in hospital stays.

### 4.5. Learning Curve and Institutional Transition

Our institutional transition occurred in a gradual and structured manner. From 2014 to 2020, cut-down cannulation represented the standard approach. Between 2020 and 2021, ultrasound guidance was introduced for femoral vessel identification while surgical exposure was still maintained, and from 2021 onward, fully percutaneous access became the routine strategy. This stepwise implementation allowed both surgeons and perfusionists to gain experience with percutaneous perfusion management and MANTA deployment before the full adoption of the technique [8]. At present, all primary surgeons in our institution were familiar with percutaneous cannulation, which has become the standard access strategy for minimally invasive procedures. In addition, as part of our residency training program, all trainees completed a three-month rotation in the vascular surgical department and routinely participated in interventional procedures in order to develop familiarity with percutaneous vascular access techniques.

Because the transition to percutaneous cannulation occurred later in the study period, the observed improvements in operative efficiency and ICU stays may partially reflect temporal changes beyond the vascular access strategy itself. Increasing surgical experience, progressive standardization of the minimally invasive workflow, refinements in perioperative care, and overall institutional maturation likely contributed to these findings; therefore, the present results should be interpreted as reflecting both the adoption of a percutaneous strategy and the evolution of an experienced minimally invasive program.

The increasing frequency of combined annuloplasty and neochordae repair during the percutaneous period indicated that the transition did not simplify the mitral valve repair strategy. On the contrary, as familiarity with visualization, atrial exposure, and leaflet handling improved, the ability to perform more complex reconstructions also expanded. This observation was consistent with contemporary evidence suggesting that minimally invasive programs matured not through simplification, but through the progressive acquisition of confidence in advanced repair techniques [10,11]. Accordingly, the reduction in operative duration likely reflected improved workflow efficiency and team performance rather than a reduced technical demand.

### 4.6. Clinical Implications

Our findings have direct implications for centers developing or expanding MIMVS programs [13,20,28,29,31,32,33,34]. Although open femoral cut-down remains appropriate in selected patients (e.g., heavily calcified femoral arteries or severe peripheral arterial disease), ultrasound-guided percutaneous access can offer clear advantages in efficiency, patient comfort, and cosmetic outcomes. The combination of smart venous cannulas and plug-based closure systems such as MANTA can enable a fully percutaneous workflow without sacrificing safety.

### 4.7. Limitations

The retrospective single-center design of this study could limit external generalizability. Selection bias may also be present, as patients in the percutaneous era were treated later in the study period and may therefore reflect increasing institutional experience, refinements in perioperative care, and progressive procedural maturation. Although all patients considered for MIMVS underwent routine preoperative CT angiography to assess suitability for peripheral cannulation, the retrospective design did not allow reliable determination of how many patients were excluded on the basis of the CT findings, including severe peripheral arterial disease. In addition, the time period was not included as a covariate in the propensity score model, and residual confounding related to the period effects cannot be excluded. Propensity score matching substantially reduced the analyzable cohort to 72 matched pairs. Although this approach improved covariate balance, it also reduced the sample size and may have limited statistical power for some secondary endpoints. In addition, the matched cohort represented a more selective subgroup of the overall population, which may affect the representativeness of the matched analysis relative to the full real-world cohort. Mortality data were incomplete for the early study years, and long-term outcomes beyond 30 days were not analyzed. Furthermore, some secondary outcomes, including re-exploration for bleeding, were analyzed descriptively and were not evaluated in dedicated multivariable models. Nonetheless, the large cohort size, the consistency of the surgical team, and the standardized institutional pathway support the internal validity of the findings.

## 5. Conclusions

In this 10-year single-center experience at Klinikum Passau, the transition from open femoral cut-down to fully percutaneous ultrasound-guided cannulation in minimally invasive mitral valve surgery was associated with shorter operative times, reduced lymphatic morbidity, and shorter ICU and hospital stays, and did not increase vascular complications or mortality. Taken together with the recent multicenter evidence, our findings can support percutaneous femoral cannulation as a safe and effective strategy in contemporary MIMVS. The observed efficiency gains, the elimination of lymphatic complications, and the expansion of reparative valve strategies could suggest that, when introduced through a structured, stepwise, team-based transition, percutaneous cannulation could represent more than a cosmetic modification and may constitute a meaningful procedural improvement in the conduct of MIMVS. However, because this transition occurred later in the study period, part of the observed benefits may also reflect institutional maturation, increasing surgical experience, and refinements in perioperative care. Centers seeking to adopt this approach should therefore prioritize the standardization of ultrasound-guided access training, structured supervision during the implementation phase, and close collaboration with vascular surgical teams.

## Figures and Tables

**Table 1:** Baseline characteristics of the patient population. Values are given as absolute numbers with % in parentheses. COPD (chronic obstructive pulmonary disease).

Baseline Characteristic	Cut-Down	Percutaneous	*p*-Value
Age, years (median [IQR])	68 [58–76]	65 [57–74]	0.031
Female sexP, *n* (%)	153 (38.9%)	59 (32.4%)	0.15
Obesity (BMI > 30), *n* (%)	91 (26.7%)	31 (17.2%)	0.009
EuroSCORE II, % (median [IQR])	1.48 [0.96–2.56]	1.21 [0.73–2.51]	0.002
NYHA class (median [IQR])	3 [2–3]	3 [2–3]	0.87
COPD, *n* (%)	40 (11.7%)	14 (7.7%)	0.20
Chronic dialysis, *n* (%)	10 (2.9%)	5 (2.7%)	0.89
Previous cardiac surgery, *n* (%)	40 (10.2%)	7 (3.8%)	0.005

**Table 2:** Types of procedures. Values are given as absolute numbers with % in parentheses.

Type of Procedure	Cut-Down	Percutaneous	*p*-Value
Mitral valve replacement	49/393 (12.5%)	18/182 (9.9%)	0.3702
Annuloplasty	287/393 (73.0%)	149/182 (81.9%)	0.0213
Neochordae implanted	221/393 (56.2%)	127/182 (69.8%)	0.0020
Annuloplasty & Neochordae	214/393 (54.5%)	127/182 (69.8%)	0.0005
Left atrial MAZE	83/393 (21.1%)	60/182 (33.0%)	0.0022
Biatrial MAZE	0/393 (0.0%)	4/182 (2.2%)	0.0098
Tricuspid valve reconstruction (TKR)	40/393 (10.2%)	20/182 (11.0%)	0.7674
ASD closure	87/393 (22.1%)	30/182 (16.5%)	0.1173

**Table 3:** Surgical results. Values are given as absolute numbers with % in parentheses.

Outcome	Cut-Down	Percutaneous	*p*-Value
Bypass time (min)	128 [107–163]	115 [98–135]	<0.0001
Cross-clamp time (min)	74 [60–95]	75 [61–94]	0.8613
Total time of the surgery (min)	242 [211–292]	210 [185–248]	<0.0001
Hospital length of stay (days)	11 [8–16]	8 [7–11]	<0.0001
ICU length of stay (days)	3.0 [2.0–5.0]	2.0 [2.0–4.0]	0.0267
Re-exploration for bleeding, total (percentage) [95% CI]	18/393 (4.5%; 95% CI 2.7–7.1)	22/182 (12.1%; 95% CI 7.7–17.7)	0.0023

**Table 4:** Access-site and device-related outcomes.

Complication	Cut-Down	Percutaneous	*p*-Value
Groin complication, total (percentage) [95% CI]	11/393 (2.8%; 95% CI 1.4–4.9)	9/182 (4.9%; 95% CI 2.3–9.2)	0.5147
Lymph fistula	17/393 (4.3%)	0/182 (0.0%)	0.0004
MANTA—bleeding, total (percentage) [95% CI]	N/A	2/182 (1.1%; 95% CI 0.3–2.3)	N/A
MANTA—ischemia, total (percentage) [95% CI]	N/A	2/182 (1.1%; 95% CI 0.3–2.3)	N/A
<30-day mortality	18/393 (4.6%)	3/182 (1.6%)	0.0962

**Table 5:** Results of the propensity score matching.

	Cut-Down Matched	Percutaneous Matched	*p*-Value
Groin complication, total (percentage) [95% CI]	2/72 (2.8%; 95% CI 0.3–9.6)	2/72 (2.8%; 95% CI 0.3–9.6)	1.0000
Bypass time (min), (median [IQR])	104.5 [89.0–123.0]	97.0 [82.0–116.0]	0.0284
Cross-clamp time (min), (median [IQR])	74.0 [61.0–89.0]	68.0 [56.0–83.0]	0.0412
Total operative time (min), (median [IQR])	244.0 [213.0–272.0]	213.0 [191.0–246.0]	0.0021
ICU length of stay (days), (median [IQR])	3.0 [2.0–5.8]	2.0 [2.0–3.2]	0.0163

## Data Availability

The original contributions presented in this study are included in the article. Further inquiries can be directed to the corresponding author.
